# Glycated albumin suppresses glucose-induced insulin secretion by impairing glucose metabolism in rat pancreatic β-cells

**DOI:** 10.1186/1743-7075-8-20

**Published:** 2011-04-06

**Authors:** Takayuki Shiraki, Yoshikazu Miura, Tokihiko Sawada, Toshie Okada, Yuhki Sakuraoka, Takashi Muto, Keiichi Kubota

**Affiliations:** 1 Second Department of Surgery, Dokkyo Medical University, School of Medicine, Kitakobayashi 880, Mibu, Shimotsuga, Tochigi 321-0293, Japan; 2 Public Health, Dokkyo Medical University, School of Medicine, Kitakobayashi 880, Mibu, Shimotsuga, Tochigi 321-0293, Japan

## Abstract

**Background:**

Glycated albumin (GA) is an Amadori product used as a marker of hyperglycemia. In this study, we investigated the effect of GA on insulin secretion from pancreatic β cells.

**Methods:**

Islets were collected from male Wistar rats by collagenase digestion. Insulin secretion in the presence of non-glycated human albumin (HA) and GA was measured under three different glucose concentrations, 3 mM (G3), 7 mM (G7), and 15 mM (G15), with various stimulators. Insulin secretion was measured with antagonists of inducible nitric oxide synthetase (iNOS), and the expression of iNOS-mRNA was investigated by real-time PCR.

**Results:**

Insulin secretion in the presence of HA and GA was 20.9 ± 3.9 and 21.6 ± 5.5 μU/3 islets/h for G3 (*P *= 0.920), and 154 ± 9.3 and 126.1 ± 7.3 μU/3 islets/h (*P *= 0.046), for G15, respectively. High extracellular potassium and 10 mM tolbutamide abrogated the inhibition of insulin secretion by GA. Glyceraldehyde, dihydroxyacetone, methylpyruvate, GLP-1, and forskolin, an activator of adenylate cyclase, did not abrogate the inhibition. Real-time PCR showed that GA did not induce iNOS-mRNA expression. Furthermore, an inhibitor of nitric oxide synthetase, aminoguanidine, and NG-nitro-L-arginine methyl ester did not abrogate the inhibition of insulin secretion.

**Conclusion:**

GA suppresses glucose-induced insulin secretion from rat pancreatic β-cells through impairment of intracellular glucose metabolism.

## Background

It has been suggested that glycated albumin (GA) is associated with the risk of complications in diabetic patients, and is used as a marker of hyperglycemia [1]. GA is an Amadori product formed non-enzymatically through the condensation reaction of glucose with reactive proteins under conditions of hyperglycemia [2,3]. Amadori products undergo further irreversible reactions to yield advanced glycation end-products (AGEs) [4,5]. Thus, Amadori products are formed through a reversible process that depends on the level of glycemia, whereas AGEs are produced irreversibly and are strong inducers of inflammation [6].

Previous studies have shown that GA itself has biological effects. Cassese et al. reported that GA induced insulin resistance in skeletal muscle cells by activating protein kinase Cα and Src [7]. Other studies have reported that GA induces the expression of proinflammatory molecules such as monocyte chemoattractant peptide (MCP-1) and interleukin-6 [8], and genes associated with fibrosis and neovascularization [9].

In general, Amadori products/AGEs trigger signaling cascades that produce oxygen free radicals, thus exposing cells to oxidative stress [10,11]. Although one previous study has indicated that glucose-derived AGEs inhibit insulin secretion from pancreatic β cells by increasing the transcription of inducible nitric oxide synthetase (iNOS) and nitric oxide [12], no investigations have addressed the effect of GA on insulin secretion from pancreatic β-cells. AGEs bind to a specific cell surface receptor, the receptor of AGE (RAGE), and exert their biological effects [13]. Because it is uncertain whether GA binds to RAGE [14], the downstream mechanism of action of GA still remains unclear. In the present study, we investigated the effect of GA on insulin secretion from pancreatic β cells and found that GA inhibits K_ATP_-channel-dependent insulin secretion.

## Methods

The present study was approved by the ethics committee of the Laboratory Animal Research Center at Dokkyo Medical University.

### Agents

GA, human non-glycated albumin (HA), α-ketoisocapronic acid (α-KIC), and 1,3-dihydroxyacetone dimer (DHA) were purchased from Sigma-Aldrich (St. Louis, MO), collagenase, D-glyceraldehyde (glyceraldehyde), and methylpyruvate from Wako Pure Chemical Industries Ltd. (Osaka, Japan), Ficoll 400 from Pharmacia Fine Chemicals (Uppsala, Sweden), Conray 400 (sodium iotalamate) from Daiichi Pharmaceutical (Tokyo, Japan), Dulbecco's modified Eagle medium (DMEM) from Nissui Pharmaceutical Company Ltd. (Tokyo, Japan), glucagon-like peptide-1 (GLP-1) from Peptide Institute (Osaka, Japan), forskolin and aminoguanidine (AG) from Sigma Chemical Co. (St. Louis, MO), NG-nitro-L-arginine methyl ester (L-NAME) from Dojindo Molecular Technologies, Inc. (Kumamoto, Japan), and disperse from Godo-Shusei Co. (Chiba, Japan). Anti-human GA neutralizing antibody, A717, was purchased from Exocell (Philadelphia, PA).

### Animals and procedures

Male Wistar rats, 8-12 weeks after birth, were obtained from Japan SLC, Inc. (Shizuoka, Japan) and housed under semi-SPF conditions. The rats were anesthetized by intraperitoneal injection of pentobarbital sodium at 50 mg/kg body weight, and their pancreatic islets were removed and subjected to collagenase digestion [15]. The islets were then separated by Ficoll-Conray gradient centrifugation [16], and individually isolated by stereoscopic microarray in DMEM containing 2% heat-inactivated fetal calf serum (FCS).

### Insulin release from pancreatic islets

The pancreatic islets were cultured overnight at 37°C in RMPI containing 10% FCS and 5.5 mM glucose, and preincubated for 90 min in HEPES-buffered solution containing 5.5 mM glucose at 37°C in 5% CO_2_-95% O_2_. Three islets were then picked up and incubated for 60 min at 37°C in 1 mL of bicarbonate buffer (pH 7.4) under three different glucose concentrations, 54 mg/dL (3 mM: G3), 126 mg/dL (7 mM: G7), and 170 mg/dL (15 mM: G15), with various agents.

According to the manufacturer, GA was produced using a method described elsewhere [17]. The physiological characteristics of the GA we used in the present study have been described previously [7,18]; it contained 195 ng CML (/mg protein), 94.9 ± 3.2 Lys modification (%), 91.6 ± 1.5 Arg modification (%), undetectable fluorescent AGE, undetectable IGF-1, and undetectable LPS. As the concentrations of the other elements were very low, we considered that the results we obtained were attributable to the biological effect of GA. In preliminary experiments, we titrated GA and HA at concentrations of 0.1, 0.5, 1.0, and 5.0 mg/mL and found that GA at the lowest concentration, 0.1 mg/mL, inhibited insulin release to a degree similar to that at higher concentrations. Also, GA has been used at 0.1 mg/mL in two previous studies [7,18]. Anti-human GA neutralizing antibody, A717, was added at concentrations of 1.25 and 5.0 μg/mL. The concentrations of other agents used in the present study were: Tolb 100 μM, glyceraldehyde 10 mM, DHA 10 mM, α-KIC 10 mM, methylpyruvate 10 mM, GLP-1 10 nM, forskolin 10 μM, L-NAME 1 mM, and AG 2 mM.

A portion of the medium was withdrawn from the incubation and appropriately diluted for the insulin assay. Insulin was measured using a double-antibody RIA kit (Eiken Chemical, Tokyo, Japan) [19].

### Measurement of intracellular free calcium in islet β-cells

Intracellular free calcium concentration ([Ca^2+^]_i_) was measured using a modification of the method of Gilon and Henquin [20], and Miura and colleagues [21]. The overnight-cultured islet cells were loaded with fura-2 for 45-60 min at 37°C in HEPES-buffered medium containing 120 mM NaCl, 4.8 mM KCl, 2.5 mM CaCl_2_, 1.2 mM MgCl_2_, 24 mM NaHCO_3_, and 10 mM HEPES (pH 7.4), containing 1 μM fura 2-AM and 5.5 mM glucose. Fura-2-loaded cells were placed in HEPES medium (37°C) containing 3 mM glucose and fixed in a hand-made chamber (fitted with a peristaltic pump for perfusion) mounted on the stage of an inverted IX 70 microscope (Olympus, Tokyo, Japan). The loaded cells were excited at 340 nm and 380 nm, the fluorescence emitted at 510 nm was captured by an intensified charge-coupled device camera, and the images were analyzed using the QuantiCell 700 system (Applied Imaging, Sunderland, UK). The changes in [Ca^2+^]_i _in single islet cells were calculated from the ratio of the fluorescence measured with excitation at 340 nm to that at 380 nm using the following equation [22]: [Ca^2+^]_i _(nM) = K_d_×(R-R_min_)/(R_max_-R)×β, where K_d _is the dissociation constant for fura-2 (224 nM), R_max _and R_min _are the ratios of the unbound and bound forms of the fura-2-Ca^2+ ^complex, respectively, and β is the ratio of the fluorescence of fura 2 at 380 nm excitation in the presence of minimum calcium and saturating calcium. R_max _and R_min _were estimated using the fluorescence intensities of fura-2 solution (1 μM) containing 10 mM CaCl_2 _and 5 mM EGTA, respectively. The fluorescent signal generated by binding of [Ca^2+^]_i _to fura-2 is not influenced by changes in the pH of the bathing solution over the range 6.0-7.05 [19].

### Measurement of cAMP content

cAMP content was measured using a modification of the method of Nelson *et al*. [23]. Pancreatic islets were cultured overnight at 37°C in RPMI with 5% FCS and preincubated for 90 min in HEPES-buffered solution containing 5.5 mM glucose at 37°C in 5% CO_2_-95% O_2_. Ten islets were then picked up and incubated for 30 min at 37°C in KRBH (0.4 mL) in the presence of stimulators. The responses were stopped by addition of 0.2 mL of ice-cold trichloroacetic acid (TCA) to a final concentration of 6%. The culture tubes were shaken, left at room temperature for 15 min, and centrifuged at 7800 × *g *for 10 min. The supernatants were thoroughly mixed with 1.5 mL of diethyl ether, and the ether phase containing TCA was discarded. This step was repeated three times to ensure complete elimination of the TCA. The extracts and cAMP standards were evaporated, treated with 400 μL KRBH, and assayed for cAMP using a RIA kit from Yamasa Shoyu (Choshi, Japan) in which the samples and standards are succinylated.

### Real-time PCR

Total RNA was isolated from islets using a Total RNA Isolation kit (Macherey-Nagel, Düren, Germany). Reverse transcription reactions were performed using a Rever Tra Ace α-First Strand cDNA Synthesis Kit (TOYOBO, Osaka, Japan). Briefly, 1 μg of total RNA, oligo dT-primer, and dNTPs were incubated at 65°C for 5 min, then 10 μL of cDNA synthesis mixture was added and the mixture was incubated at 50°C for 50 min. The reaction was terminated by adding 1 μL of RNaseH and incubating the mixture at 37°C for 20 min.

The sequences of the primers were as follows: β-actin: sense-primer 5'-agccatgtacgtagccatcc-3', anti-sense 5'-ctctcagctgtggtggtgaa-3'; iNOS: sense-primer 5'-caccttggagttcacccagt-3', anti-sense 5'-accactcgtacttgggatgc-3'.

Real-time PCR was performed using an ABI Prism 7700 sequence detector (Applied Biosystems, Warrington, UK). The PCR reaction was carried out in a final volume of 2 μL cDNA, 12.5 μL 2 × SYBR Green (Applied Biosystems), 0.5 μL of 25 nM sense and antisense primers, and H_2_O up to 25 μL. The PCR conditions consisted of 40 cycles at 95°C for 30 s and 60°C for 30 s. Samples were assayed in triplicate. Means and standard deviations were calculated from the data obtained. For each sample, at least three assays were performed. The *t *value was calculated from the mean of three different assays. The level of expression was calculated using the formula: Relative expression (t-value) = (Copy number of target molecule/Copy number of β-actin) × 1000 [23].

### Western blotting

Anti-iNOS and anti-β-actin antibodies were purchased from BD Bioscience (Franklin Lakes, NJ). Islet cells prepared as described previously were lysed with 200 μl of 0.5% (w/v) SDS, and centrifuged at 10000 rpm. The supernatants were adjusted to contain equal amounts of protein by dilution, using a BCA Protein Assay Kit (Pierce, Rockford, IL). Samples (20 μg protein) were run on 12.5% (w/v) SDS-PAGE with 10% gel and electroblotted onto PVDF membranes. The blots were blocked for 1 h with 5% (w/v) non-fat milk powder and 0.1% (v/v) Tween 20 in Tris-NaCl, then exposed to the primary antibody at a 1000-fold dilution overnight at 4°C. After extensive washing, the blots were incubated with the secondary horseradish-peroxidase-conjugated antibody (1:2000) for 2 h at room temperature. Immunoreactive bands were visualized using an enhanced chemiluminescence detection system (Amersham Life Sciences, Arlington Heights, IL). The levels of protein expression were estimated quantitatively by densitometric scanning using a Molecular Imager FX (Bio-Rad Laboratories, Hercules, CA).

### Statistics

Data were expressed as means ± SEM. All statistical analyses were performed with GraphPad Prism ver 5.0 (La Jolla, CA). Data from the two groups were compared using two-sided t-test. For Figure 3, repeated measures analysis of variance was used. Differences at *P *<0.05 were considered significant.

## Results and Discussion

### Effect of GA on insulin secretion induced by glucose, high K^+^, and tolbutamide

**Figure 1:**
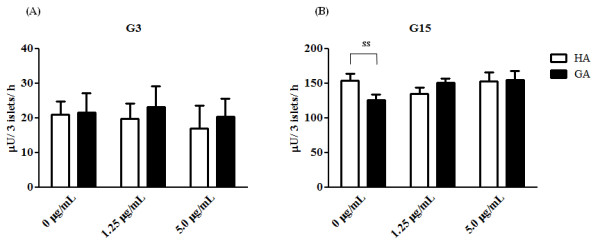
**Effect of GA on insulin secretion**. Rat islets were incubated for 24 h at 37°C with 0.1 mg/mL HA (white bar) or GA (black bar) under G3 (A) or G15 (B) conditions. Labeling of the X-axis represents the dose of anti-human GA neutralizing antibody, A717. Each bar represents the mean plus SEM of 5 separate experiments, each with n = 4. ss; statistically significant.

Insulin secretion in the presence of HA and GA was 20.9 ± 3.9 and 21.6 ± 5.5 μU/3 islets/h (*P *= 0.920) for G3 (Figure 1A), and 154 ± 9.3 and 126.1 ± 7.3 μU/3 islets/h (*P *= 0.046) for G15, respectively (Figure 1B). For G3, in the presence of 1.25 μg/mL anti-human GA neutralizing antibody, A717, insulin secretion of HA and GA was 19.7 ± 4.4 and 23.1 ± 6.0 μU/3 islets/h (*P *= 0.659), and in the presence of 5.0 μg/mL A717, the corresponding values were 16.8 ± 6.6 and 20.4 ± 5.2 μU/3 islets/h (*P *= 0.693) (Figure 1A). For G15, in the presence of 1.25 μg/mL A717, insulin secretion of HA and GA was 152.7 ± 12.8 and 150.5 ± 6.2 μU/3 islets/h (*P *= 0.194), and in the presence of 5.0 μg/mL A717, the corresponding values were 152.7 ± 12.8 and 155.0 ± 12.4 μU/3 islets/h (*P *= 0.139) (Figure 1A). Thus, the anti-human GA neutralizing antibody, A717, significantly abrogated the inhibitory effect of GA on insulin secretion.

**Figure 2:**
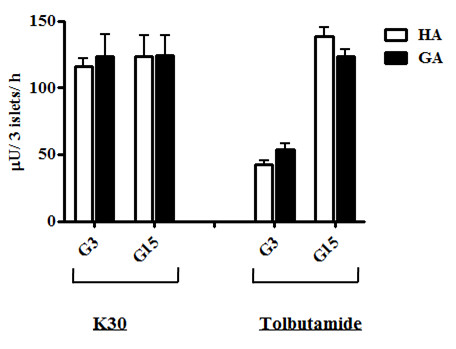
**Effects of extracellular high potassium and tolbutamide on insulin secretion**. Rat islets were incubated for 24 h at 37°C with 0.1 mg/mL HA (white bar) or GA (black bar), in the presence of 30 mM K^+ ^or 100 μM Tolb. Each bar represents the mean plus SEM of 3 separate experiments, each with n = 4.

Figure 2 shows the effect of GA on the insulin secretion induced by 30 mM K^+ ^(K30) and Tolb. K30 depolarizes the cell membrane of islet β cells without the need for K_ATP _channel closure. In the presence of K30, insulin secretion elicited by HA and GA was 116.3 ± 6.1 and 123.6 ± 16.2 μU/3 islets/h for G3 (*P *= 0.686), and 123.3 ± 15.7 and 124.3 ± 14.7 μU/3 islets/h for G15, (*P *= 0.965), respectively. Tolb promotes insulin secretion by binding to the regulatory sulfonylurea receptor-1 (SUR1) and inhibiting the K_ATP _channel current [25]. In the presence of Tolb, insulin secretion elicited by HA and GA was 42.4 ± 3.4 and 53.4 ± 4.7 μU/3 islets/h for G3 (*P *= 0.079), and 138.5 ± 6.7 and 123.4 ± 5.4 μU/3 islets/h for G15 (*P *= 0.536), respectively.

### Effect of GA on intracellular Ca^2+ ^concentration [Ca^2+^]_i_

**Figure 3:**
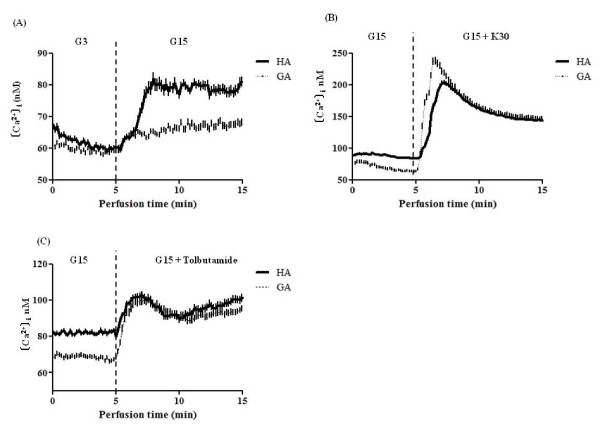
**Changes in intracellular free calcium concentration ([Ca^2+^]_i_)**. (A) Effect of GA on ([Ca^2+^]_i_). [Ca^2+^]_i _was measured under the G3 condition during the first 5 min, then under the G15 condition for the next 10 min, in the presence of 0.1 mg/mL HA (thick line) or GA (thin line). Data represent the mean plus SEM of 2 separate experiments, each with n = 4-10.. (B, C) [Ca^2+^]_i _was measured in the presence of HA (thick line) or GA (thin line) under the G15 condition. Islet cells were incubated for the first 5 min, and then in the presence of 30 mM K^+ ^(B) or 100 μM Tolb (C) for the next 10 min, in the presence of 0.1 mg/mL HA (solid) or GA (dotted). Data represent the mean plus SEM of 2 separate experiments, each with n = 4-10. ss; statistically significant.

Figure 3 shows the changes in [Ca^2+^]_i_. [Ca^2+^]_i _was measured in the presence of G3 during the first 5 min, then in the presence of G15 for the next 10 min (Figure 3A). In the presence of HA, [Ca^2+^]_i _reached a maximum level of 81.7 ± 2.0 nM at 8 min, and remained at higher level than that in the presence of GA, which was 65.2 ± 0.7 nM at 8 min, and thereafter remained lower than that in the presence of HA (*P *= 0.021). To investigate [Ca^2+^]_i _in the presence of a high extracellular potassium concentration or Tolb, [Ca^2+^]_i _was measured in the presence of HA or GA under the G15 condition for 5 min, followed by that in the presence of K30 (Figure 3B) or Tolb (Figure 3C) for 10 min. [Ca^2+^]_i _increased rapidly after 5 min when islets were cultured in the presence of G15 and K30 (*P *= 0.331), or G15 and Tolb (*P *= 0.236).

### Effect of GA on insulin secretion induced by glyceraldehyde, DHA, α-KIC, and methylpyruvate

**Figure 4:**
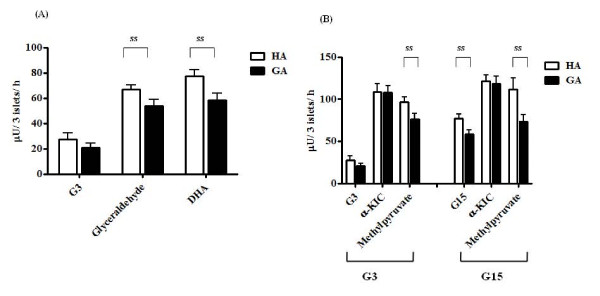
**Effects of glyceraldehyde, DHA, α-KIC, and methylpyruvate on insulin secretion**. (A) Rat islets were incubated for 24 h at 37°C with 0.1 mg/mL HA (white bar) or GA (black bar), in the presence of 10 mM glyceraldehyde or 10 mM DHA, under the G3 condition. Each bar represents the mean plus SEM of 3 separate experiments, each with n = 5. (B) Rat islets were incubated for 24 h at 37°C with 0.1 mg/mL HA (white bar) or GA (black bar), in the presence of 10 mM α-KIC or 10 mM methylpyruvate, under the G3 or G15 condition. Each bar represents the mean plus SEM of 3 separate experiments, each with n = 5. ss; statistically significant.

Glyceraldehyde and DHA enter the glycolysis pathway directly and stimulate insulin secretion [26,27]. Insulin secretion elicited by HA and GA under the G3 condition was 67.0 ± 3.5 and 53.6 ± 5.3 μU/3 islets/h in the presence of glyceraldehyde (*P *= 0.044), and 77.2 ± 5.2 and 58.2 ± 5.4 μU/3 islets/h in the presence of DHA (*P *= 0.017), respectively (Figure 4A).

α-KIC enters mitochondrial metabolism through α-ketoglutamate and increases insulin secretion [28]. Methylpyruvate is a membrane-permeable form of the mitochondrial fuel pyruvate, and also increases insulin secretion [29]. Under the G3 condition, insulin secretion elicited by HA and GA was 108.8 ± 9.4 and 107.9 ± 7.9 μU/3 islets/h in the presence of α-KIC (*P *= 0.946), respectively, and 96.4 ± 6.5 and 76.6 ± 6.6 μU/3 islets/h in the presence of methylpyruvate (*P *= 0.047), respectively. Under the G15 condition, insulin secretion elicited by HA and GA was 121.4 ± 8.0 and 118.3 ± 9.0 μU/3 islets/h in the presence of α-KIC (*P *= 0.799), respectively, and 111.2 ± 13.5 and 73.5 ± 8.2 μU/3 islets/h in the presence of methylpyruvate (*P *= 0.031), respectively.

To investigate the effect of GA on insulin production, insulin concentration in β cells was measured. The insulin content in the presence of HA and GA under the G3 condition was 1181.9 ± 72.5 and 1024.6 ± 98.6 μ Unit/3 islets, respectively (*P *= 0.222), and those under the G15 condition were 1143.6 ± 49.8 and 1040.8 ± 65.8 μ Unit/3 islets, respectively (*P *= 0.237).

### Inhibition of insulin secretion by GA is cAMP-dependent

**Figure 5:**
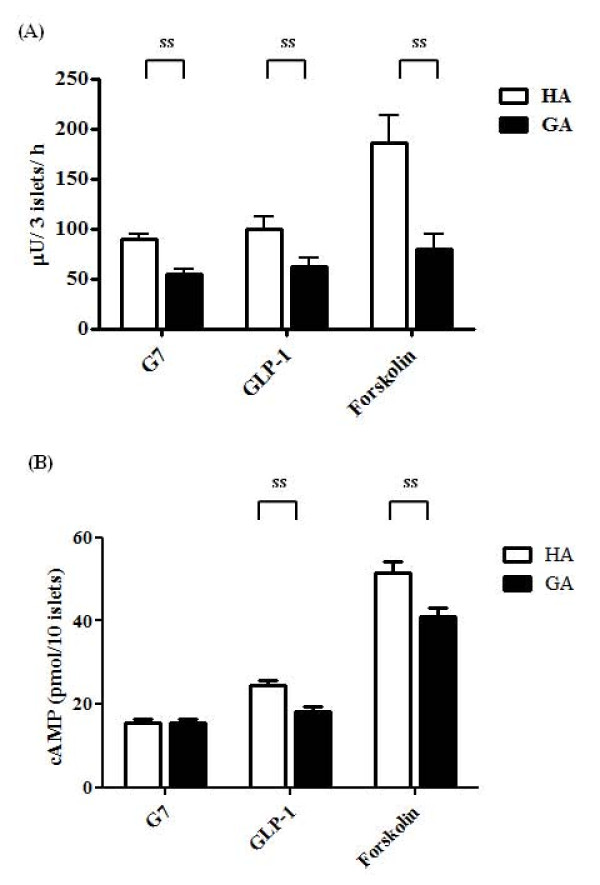
**Effects of GLP-1 and forskolin on insulin secretion**. (A) Rat islets were incubated for 24 h at 37°C with 0.1 mg/mL HA (white bar) or GA (black bar), in the presence of 10 nM GLP-1 or 10 μM forskolin, under the G7 condition. Each bar represents the mean plus SEM of 3 separate experiments, each with n = 5. (B) Dissociated islet cells were cultured overnight at 37°C in DMEM with 5% FCS. Islet cells were preincubated for 30 min at 37°C in KRBH (0.4 mL) in the presence of GLP-1 or forskolin, under the G7 condition. ss; statistically significant.

Glucose-stimulated insulin secretion is augmented by the cAMP-dependent amplifying pathway [30]. To investigate the mechanism of inhibition of insulin secretion by GA further, insulin secretion was tested in the presence of the incretin, GLP-1, and the activator of adenylate cyclase, forskolin. Insulin secretion elicited by HA and GA under the G7 condition was 99.5 ± 12.6 and 61.8 ± 9.6 μU/3 islets/h (*P *= 0.029) in the presence of GLP-1, and 185.5 ± 28.3 and 80.1 ± 14.3 μU/3 islets/h (*P *= 0.005) in the presence of forskolin (Figure 5A). Figure 5B shows the cAMP content of the islet β cells. Under the G7 condition, the cAMP contents elicited by HA and GA were 24.5 ± 1.1 and 18.1 ± 1.4 pmol/10 islets, respectively (*P *= 0.003), in the presence of GLP-1, and 51.4 ± 2.7 and 41.0 ± 2.1 pmol/10 islets, respectively (*P *= 0.018), in the presence of forskolin.

### Inhibition of insulin secretion by GA is not iNOS-mediated

**Figure 6:**
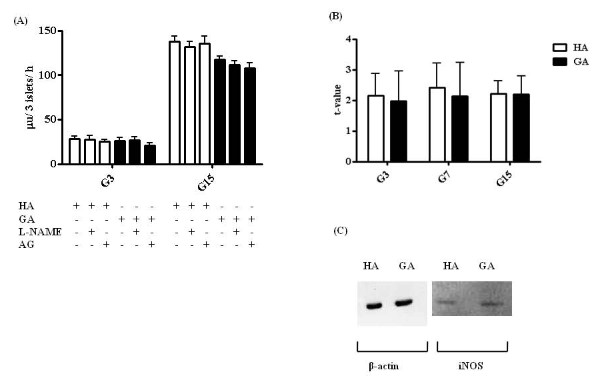
**Inhibition of insulin secretion by GA is not iNOS-mediated**. (A) Rat islets were incubated for 24 h at 37°C with 0.1 mg/mL HA (white bar) or GA (black bar), in the presence of the inhibitor of nitric oxide synthetase, L-NAME, at 1 mM, or the selective inhibitor of iNOS, AG, at 2 mM, under the G3 or G15 condition. Each bar represents the mean plus SEM of 3 separate experiments, each with n = 5. (B) Real-time PCR was performed with cDNA from RNA that had been isolated from HA- or GA-treated rat islet cells. t-value is the relative amount of iNOS-mRNA to β-actin-mRNA. (C) Rat islets were incubated for 24 h at 37°C with 0.1 mg/mL HA or GA, and the proteins isolated from islet cells were stained with anti-β-actin or anti-iNOS antibody.

Although the mechanism responsible for the suppression of insulin release by GA remains unknown, one previous study has suggested that glucose-derived AGE inhibits insulin secretion by activating iNOS, resulting in the inhibition of cytochrome *c *oxidase and ATP production [12]. Insulin secretion was therefore investigated in the presence of an inhibitor of nitric oxide synthetase, L-NAME, or a selective inhibitor of iNOS, AG [12]. Insulin secretion in the presence of HA and GA alone or with L-NAME and AG under the G3 condition was 28.2 ± 3.3, 27.6 ± 4.1, and 25.3 ± 2.2 μU/3 islets/h for HA (*P *= 0.921), and 26.3 ± 3.3, 26.4 ± 4.2, and 21.1 ± 2.8 μU/3 islets/h for GA (*P *= 0.895). The corresponding results under the G15 condition were 138.1 ± 5.7, 132.0 ± 6.1, and 135.3 ± 8.5 μU/3 islets/h for HA (*P *= 0.724), and 117.5 ± 3.8, 111.3 ± 4.4, and 107.4 ± 6.0 μU/3 islets/h for GA (*P *= 0.847). Figure 6B shows the data for iNOS-mRNA expression. The t-values of iNOS-mRNA for HA and GA were 2.2 ± 0.7 and 2.0 ± 1.0 under the G3 condition (*P *= 0.877), 2.4 ± 0.8 and 2.1 ± 1.1 under G7 (*P*< 0.001), and 2.2 ± 0.4 and 2.2 ± 0.6 under G15 (*P *= 0.822), respectively. Figure 6C shows the results of Western blotting. The relative expression of iNOS-protein to β-actin-protein in the presence of HA and GA was 0.40 and 0.42, respectively (*P*> 0.05).

The present study has demonstrated for the first time that glycated albumin (GA) suppresses glucose-induced insulin secretion from islet β-cells. As shown in Figure 3A, GA significantly decreased [Ca^2+^]_i_, and therefore the suppression of insulin secretion by GA may be due mainly to suppression of cytosolic Ca^2+ ^influx in response to glucose stimulation.

Am extracellular high potassium concentration (K30) depolarizes the cell membrane without any need for the K_ATP _channel current, and activates voltage-sensitive calcium channels; the calcium influx then stimulates insulin secretion. As shown in Figure 2, K30 abrogated the inhibition of insulin secretion by GA, suggesting that GA inhibits insulin secretion upstream of voltage-sensitive calcium channels. Tolb binds to SUR1 and keep the K_ATP _channel closed, thus inhibiting the K_ATP _channel influx of extracellular Ca^+ ^and inducing depolarization of the cell membrane, consequently inducing insulin secretion [25]. Tolb abrogated the inhibition of insulin secretion by GA (Figure 2), suggesting that GA did not affect the function of the K_ATP _channel. As both K30 and Tolb increased [Ca^2+^]_i_, (Figure 2B and 2C), the mechanism responsible for suppression of insulin secretion occurs upstream of the K_ATP _channel.

In the process of insulin secretion by β-cells, glucose is phosphorylated by glucokinase and forms glucose-6-phosphate (G6P). The G6P is further metabolized via glycolysis, to generate ATP. Glyceraldehyde and DHA are potent insulin secretagogues that enter the glycolytic pathway directly and produce ATP, resulting in insulin secretion [26,27]. In the present study, glyceraldehyde and DHA did not abrogate the inhibition of insulin secretion by GA (Figure 4A).

On the other hand, α-KIC and methylpyruvate stimulate mitochondrial metabolism and induce insulin secretion. α-KIC is a transamination partner, which enters mitochondrial metabolism through α-ketoglutamate and induces mitochondrial NADPH, thus increasing insulin secretion [28]. As shown in Figure 4B, KIC restored insulin secretion from rat β-cells, and methylpyruvate [29] did not abrogate the inhibitory effect of GA. Although the discrepancy between the effects of α-KIC and methylpyruvate is not fully understood, a previous study indicated that α-KIC not only stimulates mitochondrial metabolism but also stimulates the K_ATP _channel directly [30]. Pyruvate is an end-product of aerobic glycolysis and transported to mitochondria after oxidization to form acetyl coenzyme A (CoA), then entering the tricarboxylic acid (TCA) cycle. In β-cells, the supply of nicotinamide adenine dinucleotide (NAD^+^) from the oxidization of pyruvate is insufficient. Eto et al. reported that the NADH shuttle, including the glycerol-3-phosphate shuttle and the malate-aspartate shuttle, utilizes mitochondrial electrons to reoxidize NADH, thus playing an important role in the production of ATP in β-cell mitochondria [31]. Because, in the present study, addition of methylpyruvate did not restore insulin secretion, GA appears to also impair NADH shuttle.

GLP-1 stimulates insulin secretion by increasing cAMP [34]. Forskolin is a potent activator of adenylate cyclase [35]. Activation of adenylate cyclase/cAMP leads to the activation of protein kinase A, which in turn increases Ca^2+ ^influx to β-cells [36]. Because GLP-1 and forskolin did not restore insulin secretion and the cAMP content in β-cells, it was suggested that the pathway for amplification of insulin secretion by adenylate cyclase/cAMP was also impaired by GA treatment. We also investigated the effect of acetylcholine (ACh) on the suppression of insulin secretion by GA, and found that ACh did not elicit recovery of insulin secretion (data not shown). ACh binds to the muscarinic receptor of β-cells and activates phospholipase C-β (PLC), then stimulates release of Ca^2+ ^from the endoplasmic reticulum [37].

Zhao et al. reported that glucose-derived AGE inhibits insulin secretion by activating iNOS, resulting in inhibition of cytochrome *c *oxidase and ATP production [12]. Our results were contradictory to theirs; GA did not increase the expression of iNOS-mRNA (Figure 6A), and the inhibitor of nitric oxidase synthetase, L-NAME, and AG, did not restore insulin secretion. In Zhao's study, L-NAME and AG abrogated the inhibition of insulin secretion by glucose-derived AGE, although insulin secretion did not fully return to the normal level. As mentioned earlier, AGEs bind to RAGE, and transduce signals to downstream pathaways, including mitogen-activated protein kinases, the Janus Kinase-signal transducer and activation of transcription pathway, and phosphoinositol 3 kinase [38]. These signals result in activation of nuclear factor κB (NFκB), and increase the expression of iNOS, C-reactive protein (CRP), transforming growth factor-β, and other molecules. Although RAGE interacts with multiple ligands, it remains unclear whether GA binds RAGE or not. GA is not an AGE, and as the mechanism involved may be different, we suggest that induction of iNOS and impairment of mitochondrial cytochrome *c *does not play a major role in the inhibition of insulin secretion by GA.

## Conclusion

GA suppresses glucose-induced insulin secretion from rat pancreatic β cells through impairment of intracellular glucose metabolism.

## List of abbreviations

GA: glycated albumin: AGEs: advanced glycation end-products

## Declaration of competing interest

The authors declare that they have never received reimbursements, fees, funding, or salary from any organization that may in any way gain or lose financially from the publication of this manuscript, in the past five years, currently, or in the future.

Also, the authors do not own stocks or shares in any organization that may in any way gain or lose financially from the publication of this manuscript, either currently or in the future. The authors have not applied for any patent relating to the manuscript, and have no competing financial interests.

## Authors' contributions

All authors declare that the manuscipt was read and approved by all the authors.

T.S. Reserched the data, contributed to the discussion, and wrote the manuscript.

Y.M. Reserched the data, contributed to the discussion, and reviewed the edited manuscript.

T.S. Reserched the data, contributed to the discussion, and wrote the manuscript.

T.O. Researched the data.

Y.S. Researched the data.

T.M. Contributed to the discussion, and reviewed the edited manuscript.

K.K. Contributed to the discussion, and reviewed the edited manuscript.
